# Enhancing automated robotic services efficiency through intelligent dependent robotic computation

**DOI:** 10.1371/journal.pone.0334595

**Published:** 2025-10-22

**Authors:** Ayman Alfahid, Chahira Lhioui, Somia Asklany, Rim Hamdaoui, Monia Hamdi, Ghulam Abbas, Amr Yousef, Anis Sahbani

**Affiliations:** 1 Department of Information Systems, College of Computer and Information Sciences, Majmaah University, Al Majmaah, Saudi Arabia; 2 Department of Computer Science and Artificial Intelligence, College of Computing and Information Technology, University of Bisha, Bisha, Saudi Arabia; 3 Department of Computers and Information Technologies, College of Sciences and Arts Turaif, Northern Border University, Arar, Saudi Arabia; 4 Department of Computer Science, College of Science and Human Studies-Dawadmi, Shaqra University, Shaqra, Riyadh, Saudi Arabia; 5 Department of Information Technology, College of Computer and Information Sciences, Princess Nourah bint Abdulrahman University, Riyadh, Saudi Arabia; 6 School of Electrical Engineering, Southeast University, Nanjing, China; 7 Electrical Engineering Department, University of Business and Technology, Jeddah, Saudi Arabia; 8 Engineering Mathematics Department, Alexandria University, Alexandria, Egypt; 9 Institute for Intelligent Systems and Robotics (ISIR), CNRS, Sorbonne University, Paris, France; King Fahd University of Petroleum & Minerals, SAUDI ARABIA

## Abstract

This research focuses on enhancing the adaptability and efficacy of service robots in real-time, multi-scenario environments where diverse settings complicate the interpretation of user commands and dynamic environmental fluctuations. It introduces the Commuting Input Valuation Approach (CIVA), which combines transfer learning with a flexible state transfer system to improve robot response rates, minimize unnecessary actions, and enhance learning efficiency across varied environments. The main contributions are (i) a continuous training framework for robots, (ii) a method for sharing information between response and learning stages, (iii) transfer learning approaches suited for both short and long inputs, and (iv) an adaptive reaction rate calculation that accounts for real-time conditions. CIVA was evaluated on 32 interactive tasks using the Daily Interactive Robot Manipulation (DIM) dataset, containing 1,603 dependent and 1,751 independent commands. Performance was assessed relative to baseline robotic models utilizing criteria including task completion duration, input interpreting failure rate, command-to-action efficiency, reaction successful rate, and learning velocity. In a targeted screw-loosening assessment comprising 120 input commands over 70 seconds, CIVA demonstrated a 35% reduction in task completion duration, a 42% decline in interpretation errors, a 28% enhancement in instruction-to-action efficiency, a 45% augmentation in response success rate, and a 50% increase in learning velocity relative to baseline measurements. The findings indicate that CIVA can enhance human-robot interaction and task performance; nevertheless, additional validation is necessary to verify reproducibility and generalization across diverse real-world contexts.

## 1. Introduction

Automation allows robots to do activities without human involvement. This improves operational efficiency, reduces human workload, and optimizes service delivery in industrial and service sectors [1]. Human interactions with small service robots have been studied to uncover behavioral patterns, response, and engagement characteristics. The study examined user behavior and robot responsiveness using observational research and controlled tests [2]. The goal was to improve robot design for adaptive reactions. An examination of how robots have affected farming, manufacturing, and service jobs was conducted to better comprehend their effects. This study used statistical analysis of sectoral employment data and labor shift modeling to investigate how robots alter job and task distribution [3]. Interactive robotic systems have been studied using stochastic simulation and modeling techniques to optimize operating efficiency, safety, and performance. Probabilistic simulation and scenario-based modeling were used to forecast and regulate robot interactions in uncertainty [4]. Modern computer vision methods like reconstructed convolutional neural networks have been used to recognize objects in shifting environments. The strategy used street view datasets to build deep learning models for real-time object detection [5]. Our goal was to improve robots’ autonomous decision-making.

Multimodal household robot systems improve human-robot communications and task performance. These systems help expressively impaired users. The goal was to create a more accessible robotic support system using speech, gesture and facial recognition and task-planning algorithms [6]. To increase workflow efficiency and maintain operations throughout the epidemic, takeout and catering businesses have utilized trained robots to automate service. Supervised instruction and process automation were used to improve service delivery within restrictions [7]. Computing vision and soft computing methods have been combined to improve robot navigation and obstacle avoidance. Improvements were made to improve autonomous mobility. Sensor integration, path planning algorithms, and real-time decision-making were used [8]. Robot anatomy, controls, and optical systems have been optimized via modular design automation. To improve task adaptability and efficiency, modular design framework and system-level simulations were used [9].

Research on gender implications in human-robot interactions examined how gender matching influences service quality and user satisfaction. Experimental designs and behavioral analytics were used to improve personalized service [10]. Precision robotic deburring with simultaneous registering and machining improves industrial quality, precision, and efficiency. CAD-based modeling, sensor calibrating, and automatic machining control were used [11]. To improve service and comfort, service robots have been studied for their ability to reduce client embarrassment during sensitive conversations. The process included scenario-based testing and user surveys [12]. Reinforcement learning improved robot adaptation and task performance in affordance-based human-robot interaction research. Iterative learning techniques and simulation-based training achieved this [13]. We used meta-reinforcement learning and model predictive control to improve mobile robot adaptability and reduce errors in dynamic conditions. Methodology included modeling of predicted outcomes, reward-based learning, and incremental policy optimization [14].

One-shot domain-specific imitation learning is used for robotic pouring operations to rapidly adapt to several domains. Transfer instruction and progressive educational frameworks were used for job generalization [15]. AI-powered human-robot cooperation (HRC) frameworks have been studied to improve consumer experience and operational efficiency [16]. The method included literature reviews, case studies, and comparative analysis. Configurable process control techniques optimize industrial robotic services via adaptive control techniques and simulation-based testing [17]. Flexibility in work execution and monitoring has achieved this. The processing of natural languages, semantic mapping, and hierarchy task planning have been studied to increase service robot responsiveness [18]. Task knowledge and command-triggered execution have been studied. Human-small service robot interactions were studied using observational research and performance assessments [19]. These observations aimed to foster adaptive learning. Researchers compared service robots to human personnel in terms of client behavior, satisfaction, and service outcomes using behavioral experiments and controlled trials [20].

Complex engineering, finance, and leadership optimization problems with big search spaces, nonlinear restrictions, and competing goals are hard for traditional methods to solve. Although many meta-heuristic algorithms have been created, none of them work well in all types of problems. It is also not clear how fast they converge, how much they cost to run, or whether they can do global or local searches. As a result of their ability to lower the amount of work needed to run computers and balance research and exploitation in large-scale optimization tasks, these algorithms need to be formally reviewed. Referenced study looks at meta-heuristic algorithms, pointing out their pros and cons and showing how they can be used in different areas to assist researchers and professionals in picking the best optimization strategies [21] or (Moshayedi et al., 2024a).

Standard approaches struggle to solve complex, nonlinear, and large-scale optimization issues in many fields. Many meta-heuristic algorithms have limitations such premature convergence, parameter sensitivity, and poor scalability in high-dimensional spaces, hence no single solution works for all applications. Even though meta-heuristic methods have been introduced. Due to this difficulty, a deeper understanding of how meta-heuristic approaches behave in different problem settings, particularly in terms of convergence, exploration–exploitation balance, and resilience, is needed. The paper compares population-based and other meta-heuristic algorithms to highlight their merits, weaknesses, and performance trade-offs. The study also seeks to highlight open issues and future directions to help researchers and practitioners choose or build better optimization solutions [22] or (Moshayedi et al., 2024b).

Environmental uncertainties and restrictions in transferring simulation results to real-world applications make service robot control and performance difficult. Robot motion deviates and is inefficient when traditional controllers like PID struggle to maintain robustness throughout circular, elliptical, spiral, and octagonal trajectories. The requirement for optimization approaches to tune controller parameters and ensure reliable navigation and manipulation in practical circumstances arises from this constraint. FOODIEBOT aims to design and develop a vision-enabled service robot that can manipulate and navigate objects, validate its performance in simulation and real environments, and evaluate optimization algorithms for calibrating control parameters for improved accuracy, speed, and adaptability across diverse trajectories [23] or (Moshayedi et al., 2024c). The main research objective of this work are outlined below:

- Iterative training helps service robots perceive and respond to a variety of human inputs in dynamic, moving environments.
- With a unique conversion factor and transfer learning algorithms that can handle varying input lengths, you can optimize knowledge transfer between the response and learning states.
- Use adaptive reaction rate calculation to improve robotic interaction. The computation should accommodate for environmental disruptions and real-time context change.
- Validating and generalizing the process across many robotic manipulation methods ensures reliable and seamless service robot performance in various situations. The Commuting Input Valuation Approach (CIVA) has four concise study goals.

The main contributions of this work are outlined below

- Robots can adapt to changing environments in real time with this continuous iterative training system.
- A new way to exchange states that uses a conversion factor to move information from the response stage to the learning stage.
- There are transfer learning methods that can handle both short (low-length) and long (high-length) input commands, which makes conversion factor optimization better.
- An adaptive response rate calculation that takes into account changes in the surroundings and changing conditions in real time to improve the timing of interactions.

A full experimental study using 32 interactive tasks from the Daily Interactive Manipulation (DIM) dataset, with 1,603 dependent and 1,751 independent commands, and focused tests like a screw-loosening task showed big improvements in how quickly the tasks were finished, how often mistakes were made in interpreting the instructions, how well the responses were made, and how fast the models learned compared to baseline models.

The rest of the paper is followed by Section 2, describing the related works with the latest literature review. Section 3 discusses the proposed Commuting Input Valuation approach in detail. Section 4 gives the results and discussion obtained from the simulation with different metrics. Section 5 finally concludes the outcome of the proposed approach.

## 2. Related works

Speech recognition and multimodal interaction systems to improve robot command interpretation and enable genuine human-robot communication (Qin et al., 2023 [6]; Xi & Zhu, 2023 [18]). Text-to-action conversion methods turn spoken or written instructions into robot actions, enhancing robot adaptability in task execution (Xi & Zhu, 2023 [18]). Robots can also comprehend situational cues with contextual awareness, thereby improving interaction quality (Gómez & Miura, 2021 [24]). Service robots react to dynamic environments through online learning and exploration strategies, enabling real-time adaptation (Rayyes et al., 2020 [25]). Furthermore, adaptive planning and decision-making frameworks strengthen robots’ ability to handle complex service scenarios (Tolba & Al-Makhadmeh, 2022 [26]). Finally, efficiency optimization studies emphasize calibration and algorithmic improvements to enhance accuracy, processing speed, and task performance for seamless operation in diverse environments (Chen et al., 2024 [27]; Selami et al., 2023 [28]).

### 2.1. Speech recognition systems for robot command interpretation

Chen et al. [27] proposed a hybrid evolutionary scheme (HOE) model for robot calibration. The main aim of the model is to evaluate the efficiency and calibration of the robots. The proposed model uses an evolutionary computing (EC) algorithm that analyzes the performance loss of robots. The proposed model identifies the robots’ high calibration accuracy, enlarging the operations’ feasibility and quality range. Liu et al. [29] developed an active object detection (AOD) using a deep Q-learning network-based approach for service robots. The learning approach is mainly used to analyze the valuable status data of the robots during training. The robots’ current state and status are evaluated, providing a proper viewpoint for AOD. More energy is required to detect the actual types and classes of objects. The developed method maximizes the precision of AOD in service robots.

### 2.2. Text-to-action conversion in robot interfaces

Rayyes et al. [25] proposed a new online learning method for robots to reduce the high sample complexity in robots. The proposed method mainly accelerates the robots’ online learning skills and range. The proposed method is a data-driven model that minimizes the complexity and latency in the online learning process. Internet-driven exploration is utilized in the method, which provides effective signals to the robots during the learning period. Hao et al. [30] designed a meta-residual policy learning (MRPL) for robot skill adaptation. The main aim of the model is to reduce the cost of policy in learning and adaptation. The designed MRPL model uses a knowledge fuse technology to learn the exact condition of the policies. A hand-engineered controller is implemented to evaluate the embedded tasks in the policy. The designed model enlarges the performance level of robots in the skill adaptation process.

### 2.3. Contextual understanding in human-robot communication

Zhou et al. [31] introduced a model-based actor-critic learning algorithm to learn interactive skills in a complex interactive environment. The actual role of the algorithm is to reduce the complexity during interaction services with unknown environments. It is used as a safety-learning strategy that learns the impedance control for the data-transferring process. The introduced algorithm enhances the effectiveness and performance ratio of the robots. An improved version of [27] is proposed by Salami et al. [28] called a 3D positioning and posture (3DPP) sensor-based robot calibration method. The 3DPP sensors are used here to analyze the exact distance and location for the calibration process. The proposed method is widely used to reduce robot task and workload ratio in various industries. It is a time-consuming method that enhances the feasibility level of the robot calibration process.

### 2.4. Real-time environmental adaptation in robots

Őrsi et al. [32] studied a robot request accepting contradiction via human instruction. Robots’ exact level of interdependency and attitudes are analyzed for the human-robot interaction process. The analyzed information reduces the computational cost in the interaction process. The studied area provides feasible data to increase the request-accepting ratio of the robots during interaction services. Park and Han [33] developed a reinforcement learning (RL) framework using multimodal advantage function (MAF) in robot learning. The developed framework is used to estimate the accurate advantage of robots in the learning process. It is also used to enhance the robots’ sample-efficiency range, eliminating unwanted routing traffic on robots. Experimental results show that the developed framework maximizes the accuracy of the estimation process.

### 2.5. Online learning methods for dynamic environments

Yin et al. [34] proposed a dynamic vector hybrid genetic algorithm (DVHGA) for robot cloud service selection. It is a two-stage algorithm used to update the functional capabilities of the policies. The algorithm selects the services based on necessity and priority, which enhances the robots’ quality of service (QoS) ratio. The proposed DVHGA enlarges the robots’ performance and feasibility range. Chiang and Chou [35] explore the service quality of robots for different levels of intimacy. The new environmental psychology methodology is employed in the model, which analyzes the different levels of intimacy of the robots. It also evaluates the high intimacy level of the robots to improve the QoS in providing services to the users. The proposed model minimizes the detection latency, improving the networks’ reliability level.

### 2.6. Adaptive planning and decision-making in changing scenarios

Özer and Erden [36] developed a behavioral model for social robots using a 3D design structure matrix (DSM) model. The actual goal of the model is to enhance the systematic development of social robots. The developed model provides various alternative routes to perform tasks for the users. The robots’ exact behavioral patterns are evaluated, reducing the improvement process’s complexity. Wang et al. [37] designed a multi-granularity service composition model for industrial cloud robots. It utilizes the coarse-grained services presented in multi-functional robots for the industries. The operations provide certain advantages to improve the production range of the sectors. Compared with other models, the designed model maximizes the functional capabilities of the industrial robots.

### 2.7. Efficiency optimization in service robots

To enhance the service range of indoor robots, Zhao et al. [38] developed a new asymmetric multiscale and cross-modal fusion network (AMCFNet) for semantic segmentation. A semantic aggregation module (SAM) is implemented in the model to analyze the spatial features for the improvement process. The SAM eliminates the unwanted latency, which causes damage in the QoS enhancement process. The developed model is mainly used to enhance the performance and QoS range of the robots. Gómez and Miura [24] proposed a modular interactive computation scheme (MICS) for Internet of Things (IoT) assisted robots. The MICS is mainly used to identify the exact resource utilization ratio of IoT-assisted robots. The proposed scheme also validates the performance and feasibility ratio of the robots. The proposed scheme reduces the overall computational complexity ratio when providing services. Tolba and Al-Makhadmeh [26] introduced an ontology-based knowledge management for human-robot interaction. The introduced system uses specific modules to manage the knowledge and reasoning level of the robots. Verbal interactions are provided to the robots to help them understand the exact content/meaning of the task requests. The introduced system enhances the feasibility and robustness range of the service robots. Sheron et al. [39] proposed a projection-dependent source processing procedure to minimize the misidentification of target objects identified by robots with human interaction. The practical training phase discusses the possible dimensions for identifying intersection points in real-time applications and preventing errors. The robot’s training environment is configured to detect the object’s position and motion in space. The study identifies the learning state and condition using recurrent analysis from the acquired images in 3D format. The introduced system provides an improved recognition ratio, reduced time complexity, and minimal error.

### 2.8. AI and robotics-driven automation in industrial and operational systems

Automatic robotic solutions are proposed in this study as a means of enhancing precision molding in the rubber manufacturing industry. The paper’s objective is to improve accuracy, consistency, and efficiency while simultaneously minimizing waste. The issue that is being addressed is the inefficiency and variability that are inherent in the conventional manual molding procedures. The results demonstrate an increase in throughput, a reduction in mistakes, and an improvement in molding precision. Among the limitations are the high costs of implementation, the complexity of integration, and the requirement for expert operators. Additionally, additional research is required for the development of various materials and wider use Tejani, J. G. [40].

A proposal is made in this work to directly incorporate safety requirements into smart robots in order to take advantage of their computational and collaborative capabilities while also guaranteeing that they comply with the law. Regarding the deployment of advanced autonomous robots within the European Union, the regulatory and liability gap is the primary issue that is addressed. Based on the findings of the study, incorporating safety procedures that are enforceable can reduce the likelihood of legal hazards and increase trust. Among the limitations include the theoretical focus, the absence of empirical validation over a wide range of industrial settings, and the difficulties associated with standardizing compliance across a variety of geographical jurisdictions.ecellio Segate, R [41].

For the purpose of transforming the automation of the construction industry and increasing labor productivity, the study suggests the implementation of artificial intelligence and robotic technology. The principal issue that is being addressed is the ongoing inefficiency and labor-intensiveness that is present in the construction procedures. According to the findings of the study, making use of robotics that are driven by artificial intelligence can increase operational efficiency, minimize reliance on manual labor, and speed up project timeframes. The high implementation costs, the impediments to technology adoption in underdeveloped nations, and the limited long-term empirical studies on productivity increases across a variety of building projects are some of the limitations Faheem, M. A [42].

The literature evaluation has exposed many serious holes in studying service robot flexibility. Suboptimal performance and poor flexibility are common outcomes of current techniques’ inability to efficiently process and respond to different inputs from users in heterogeneous situations. Many current systems can’t adapt their learning and response processes on the fly to various user commands and changes in the environment. Additionally, there are limited efficient methods for transferring information between different robot states, such as the response and learning phases, to enhance performance. When it comes to optimizing transition among several types of input commands, there is a lack of research on how robot adaptability systems may include sophisticated machine learning methods like transfer learning. Also, the problem of enhancing learning rates and adaption speeds while reducing robot responses that aren’t necessary is generally ignored in the existing literature. Taken as a whole, these deficiencies show how service robot training and functioning in varied, real-world contexts has to be more thorough, flexible, and effective.

In contrast to earlier methods that rely on intrinsic motivation, static residual policy fusion, or psychological state estimate, CIVA presents a quantitative and modular approach to real-time robotic adaptation. The input valuation layer allows for the contextual modification of incoming data, and the state conversion mechanism uses a predetermined deviation threshold to quantify the requirement for behavioral shift. Furthermore, unlike previous research, CIVA’s policy fusion process is adaptively weighted using a *λ* blending coefficient, and it is further improved using a fuzzy decision system that deals with ambiguous environmental signals. Table 1 provides a comparative examination of the CIVA methodology that has been proposed in comparison to the methods that are currently in use. In contrast to earlier models, the CIVA model integrates multiple features, including increased transfer learning, quantified dynamic state conversion, and adaptive weight-based input valuation. Additionally, it incorporates λ-adaptive policy blending and fuzzy logic in order to offer the capability of flexible decision-making. In general, CIVA consistently exhibits greater real-time flexibility and robustness across a wide range of dynamic settings.

**Table 1:** Comparative analysis of existing methods versus the proposed CIVA approach.

Feature/ Method	[21] Interest-driven	[22] Transfer + Residual	[29] Psych. Behavior	CIVA (Proposed)
Input Valuation Mechanism	✖	✖	✖	✅ Adaptive weight-based
Dynamic State Conversion	✖	✖	Partial (emotional)	✅ Quantified deviation Δs
Transfer Learning Support	✅	✅	✖	✅ (Enhanced via fuzzy blending)
Policy Fusion Adaptability	✖ (fixed strategy)	Partial (static residual)	✖	✅ λ-adaptive blending
Real-Time Environmental Adaptation	✖	✖	Partial	✅ Core functionality
Fuzzy Logic Integration	✖	✖	✖	✅ For decision flexibility

## 3. The proposed commuting input valuation approach

The suggested solution addresses service robot adaptive input-response issues. The problem setting is defined, and the experiment data are briefly described. User commands are modeled, and a state exchange mechanism enables dynamic context switching. Transfer learning optimizes conversion factors, enhancing adaptability. Adaptive response rate computation optimizes interaction timing, while input extraction and feature analysis gather relevant data. The flexibility of service robots is continually monitored. Final integration creates a seamless workflow for efficient and intelligent robotic performance.

### 3.1. Problem context and definition

The rapid deployment of service robots across various industrial, commercial, and domestic environments highlights their increasing importance. However, these robots often face significant challenges in adaptability, particularly in heterogeneous settings where user inputs and environmental conditions vary dynamically. Traditional robots may not effectively process or respond to diverse scenarios, leading to inefficiencies and reduced user satisfaction. CIVA iteratively refines the robot’s ability to prioritize and respond to user inputs. The state exchange mechanism facilitates efficient knowledge transfer between response and learning states. Then, transfer learning optimizes the ability of the robot to convert between low-length and high-length inputs, enabling it to handle a wide range of instructions.

The proposed study seeks to accomplish essential goals in robotic service adaptation while introducing many new concepts. Iteratively training robots to understand varied scenarios in real-time heterogeneous surroundings is one of the primary innovations. The approach is called the Commuting Input Valuation Approach (CIVA). When presented with several instructions and responses, this strategy successfully identifies and improves the performance of less adaptive service robots. The use of a conversion factor is novel in this research to promote knowledge transfer between the response and learning phases is another noteworthy innovation. This mechanism is based on a state exchange. To improve the efficiency of robotic operations, the research also uses transfer learning to handle conversion factors between low-length and high-length inputs. We provide a new approach to computing and boosting reaction rates that accounts for ambient elements and potential disturbances in real time.

Extending the experimental evaluation to incorporate at least three to five different robotic actions from the Daily Interactive Manipulation (DIM) dataset—for example, opening drawers, twisting bottle caps, sorting objects, and wiping surfaces—with varying degrees of sensory and motor complexity would improve the results’ generalizability. This more comprehensive evaluation would show how well the suggested CIVA framework works in various manipulation scenarios. The “conversion factor” measure, which should be defined as the ratio of genuine system-triggered state transitions (where calculated deviation Δ s exceeds a predetermined threshold θ s) to total environmental state changes, should also be explicitly stated. A more significant conversion factor indicates more immediate adaptation to changing conditions, whereas a lower number suggests stability or delayed reactions. The statistic becomes more meaningful and demonstrates CIVA’s real-time decision efficiency when these values are contextualized and interpreted across various activities.

### 3.2. Data briefing

The data used in this article is collected from the Daily Interactive Manipulation (DIM) dataset [43] that verifies robot position, orientation, and other mechanical movements. This dataset provided 32 interactive robot tasks under 1603 dependent and 1751 independent instructions. The robot’s adaptability is estimated through zero-difference in final and actual robot motion. From the 32 actions, we utilize 1 action under different commands (say 120 inputs) for a maximum of 70s service time. This data is collected from the “DIM” dataset [43], which verifies robot position, orientation, and other mechanical movements. In particular, Table 2 describes the experimental setting of the research study for the adaptability of a robot. In the reference representation below, the data modeling is presented.

**Table 2:** Experimental variables and parameters for evaluating robot adaptability performance.

Variables	Description
Tasks	32 Interactive robots
Instructions	1603-dependent 1751-independent
Inputs	Out of 120 inputs (1 screw loosening action is focused)
Service time	Max 70seconds
Data Sequence Format	Input number, with x and y coordinates, x-variation, y-variation, orientation, and torque

**Fig 1:**
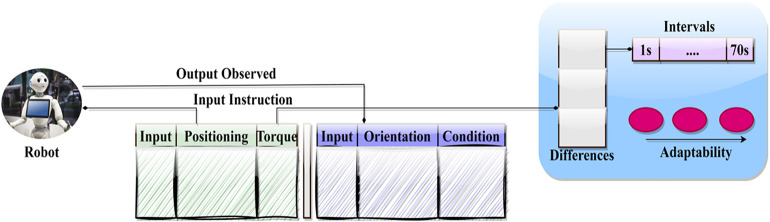
Robot action representation.

Within the context of the situation described above, the action of the baking robot loosening the screw is taken into consideration. The sequence of data is structured as follows: [input-no, (x-y coordinates), (x-variation, y-variation), orientation, and torque]. The adaptability measure of the instruction feed is defined by this variation, which is based on the output that was seen. With the input valuation serving as the defining factor for the conversion, the observation is sequenced for the 1970s under the task completion heading. For this input valuation, transfer learning defines several different states (see to reference Fig 1). The flow of the experimental process that was used to evaluate the adaptability of service robots is depicted in Fig 1. It demonstrates how the robot is given input instructions, which include positioning, torque, orientation, and condition, and how the observed outputs of the robot are compared over a range of time intervals, which can range from one second to seventy seconds. To evaluate the robot’s adaptability in real-time dynamic situations, these changes in responses between intervals are examined.

Each of the baseline procedures was carried out under the same experimental settings and with the same experimental data to guarantee fairness and consistency in comparison. The evaluation consisted of 32 interactive robot tasks, comprising 1,603 dependent instructions and 1,751 independent instructions. There were a total of 120 different input actions that were taken into consideration, with the “screw loosening” scenario being focused on exclusively. A maximum service time of seventy seconds was used to measure the total amount of time spent on each task. Input numbers, x and y coordinates, variations in both axes, orientation, and torque values were all included in the data sequences. This provided a comprehensive set of parameters that could be used to validate the adaptability of the robot and ensure that all testing settings were equal for all techniques.

### 3.3. Input modeling

This approach aims to maximize the service adaptability of robots in real-time applications. Consistent and periodic training on user inputs like voice, text, etc., result in less adaptability services due to unnecessary responses. In this valuation, the probabilistic of robotic service adaptability is computed as


$$\begin{array}{c} \text{max}\sum {\mathrm{\backslash nolimits}}_{i=A_{robs}}\sum {\mathrm{\backslash nolimits}}_{j={Input}_{val}}\rho {\left({Service}_{\alpha T},\;j\right)}^{\alpha T} \end{array}$$ (1)

Such that,


$$\begin{array}{c} \sum {\mathrm{\backslash nolimits}}_{i=A_{robs}}{\left({Service}_{\alpha }\right)}_{T}={Iterative}_{tr} \end{array}$$ (2)

$$\begin{array}{c} \prod {\mathrm{\backslash nolimits}}_{i=A_{robs}}{\left({Service}_{\alpha }\right)}_{T}=\prod {\mathrm{\backslash nolimits}}_{i=A_{robs}}\left(\sum {\mathrm{\backslash nolimits}}_{j=T}\left({\left({Service}_{\alpha }\right)}_{T}-\text{1}-\left[\frac{{\left({Service}_{\alpha }\right)}_{j}}{\sum {\left({Service}_{\alpha }+\nabla_{T}\right)}_{i}}\right]\right)\;\right) \end{array}$$ (3)

In equation (1), the variable $\;\rho \left({Service}_{\alpha t},\;j\right)$ used to represent the probability of service adaptability of robots is computed through user input valuation $\;{Input}_{val}$ and iterative training ${Iterative}_{tr}$ from the automated robotic operations *A*_*robs*_. The maximum service adaptability of *α* = 1 is to achieve maximum response from the robots based on the training level. Where the variables $\;\nabla_{T}$ and *T* represents the previous knowledge of response and training level. Contrarily, if *A*_*robs*_ and *T* is not constant due to *c* as  α∈[0,1] is the maximum input to increase its training level. Therefore *α* = 1 is not recurrently analyzed, and this condition results in less response and learning states. This problem is referred to as adaptability-less robotic operations. The assisting input valuation and proposed approach jointly produce maximum service adaptability. The iterative input modeling definition is presented through Algorithm 1.

Algorithm 1. Iterative Input Modeling

Input: $A_{robs}\;//robot\;services\;limit\;$

Output: α//optimized service

1. Initialize:

$for\;i=\text{1}\;to\;A_{robs}\;do$

2.Define Objective:

$for\;i=\text{1}\;to\;A_{robs}\;do$

Repeat

3. Iterative Computation:

Compute Service Product:

$\begin{array}{c} ompute\;\prod_{i=A_{robs}}{\left(service_{\alpha }\right)}_{T}\;until\;A_{robs}=max\; \end{array}$

**Check Condition:**

$if\left[{\left(service_{\alpha }\right)}_{T}=Iterative_{tr}\right]\;then\;//condition\;check$

      $\begin{array}{c} \;\;\;\;\;\;\;\;\;\;\;\;Input_{val}=Input_{val}+\text{1};\\ \alpha =\frac{Input_{val}}{\nabla_{T}}; \end{array}$

Re-evaluate Input Modeling:

if α≠1 then

goto step 3;//Repeat for new i/p modeling

end if

4. Termination:

end if

Until convergence or maximum iterations are reached

5. End Loop

end for // closing conditions & Loops

This method improves robots’ service flexibility by repeatedly honing input valuation. Vital signs consist of: *A*_*robs*_ the limit of robot services being evaluated, *α* represents the optimized service adaptability factor, $\rho \;\left(service_{\alpha T}\right)$ is the probability of service adaptability for input j at time T and $\nabla_{T}$ denotes the response and training level prior knowledge. The algorithm tests a service’s adaptability to see if it satisfies the iterative training criterion. If this doesn’t happen, it will keep trying until it reaches convergence or the maximum number of iterations.

### 3.4. State exchange mechanism

The main goal of this research is to educate robots on user inputs like text and speech regularly and consistently to modify their services better in real-time applications. The goal is to make robots better at comprehending and responding to different user commands through improved interactive responses. One crucial objective is to solve the problem of service robots that aren’t as adaptive, which is especially problematic in diverse locations where robots must comprehend varied situations. To accomplish this, the work is concentrated on improving the robot’s overall efficiency by maximizing the conversion between the response and learning phases. Taking into account aspects like disruption rates in voice/text orders is another way to make robots more resistant to changes in real-time surroundings. An additional important goal is to enhance training efficiency by decreasing the number of needless replies using mathematical probability and transfer learning models. Last but not least, the research aims to generalize its technique to different robotic activities and situations while emphasizing a specific activity (like screw loosening) for robots. The overarching goal of these advancements is to improve robotic efficiency and adaptability in complex, real-world settings.

Such issues are addressed in automated robotic operations to improve the robot’s service adaptability to the environment. Several unique robotic service flexibility ideas are presented. This breakthrough, CIVA, iteratively trains robots to handle various circumstances in real-time heterogeneous environments. It works well for service bots that have difficulty with multiple orders and answers. State exchange uses a novel factor of conversion to transport knowledge between reaction and development stages, another novelty. Transfer learning processes factor in conversion among low- and high-length inputs, boosting robotic service efficiency. A real-time environmental component and disturbance-based reaction time computation and enhancement approach are also introduced. The innovations aim to boost real-world robot adaptability, effectiveness, and resilience. The diagrammatic illustration of the proposed approach is given below.

**Fig 2:**
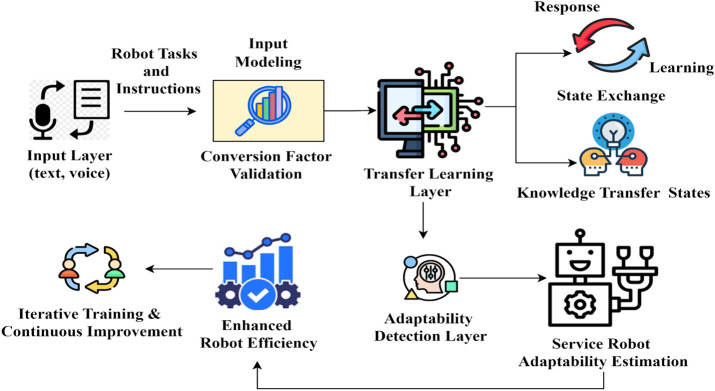
Diagrammatic illustration of the proposed approach.

Fig 2 illustrates the proposed approach of CIVA, and the less robotic service adaptability is addressed in the heterogeneous environment that is taken according to whether the maximum input to response conversion factor is accounted for increasing its training level. In this manner, if the response is referred to as 1 and the learning state is referred to as 0. Based on this valuation, the conversion factor in which the knowledge between the states is exchanged and is symbolized as $\;{Convs}_{fact}$ for before and after the adaptability of robotic services. In particular, if 1 shows which state the conversion factor is considered and the remaining states, 0 shows the states exchanged before the conversion factor is accounted for. Accordingly, the states can be exchanged in four ways:

1. The response to learning states before conversion factor consideration $\;\left({Service}_{\alpha }=\text{0},\;{Convs}_{fact}=\text{0}\right)$,
2. The response to learning states after conversion factor consideration $\;\left({Service}_{\alpha }=\text{0},\;{Convs}_{fact}=\text{1}\right)$,
3. Learning states to respond before conversion factor consideration $\;\left({Service}_{\alpha }=\text{1},\;{Convs}_{fact}=\text{0}\right)$ and
4. Learning states to respond after conversion factor consideration $\;\left({Service}_{\alpha }=\text{1},\;{Convs}_{fact}=\text{1}\right)$.

**Fig 3:**
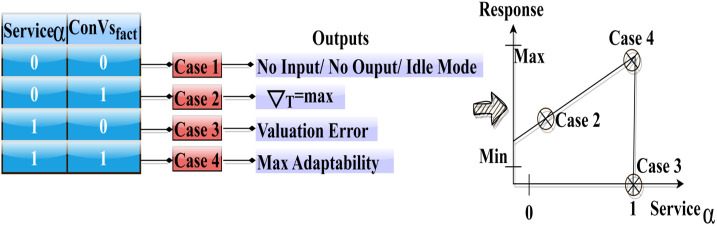
State exchange possibilities.

The input valuation of $\;{Service}_{\alpha }\times {Convs}_{fact}$ robotic service adaptability and conversion factor is computed using the transfer learning is the total efficiency of the automated robotic services. Fig 3 presents the state exchange possibilities for the above 4 cases with the outputs.

The response for a case is analyzed based on $service_{\alpha }$ and $convs_{fact}$ exchange between the states. The input modeling achieves maximum training level for cases 1 and 3, retains $\;\nabla_{T}$ for case 2 and achieves less iterates for case 4. Therefore, the knowledge between the states is swapped for cases 1 and 3 frequently. This impacts the *A*_*robs*_ by reducing the response factor regardless of the $\nabla_{T}$ (Fig 3). The use of the CIV approach enhances the automated robot’s required services based on the conversion of two states performed to the different scenarios, thereby achieving a highly interactive response for the robots. In particular, the state’s conversion of all aspects of improving the robotic service adaptability is based on consistent training. However, due to the lack of support for robotic service adaptability in developing nations, it is challenging to train robots to understand various scenarios and provide multiple instructions and responses.

To overcome this issue, we utilize the proposed approach developed to improve the robots’ interactive response and service adaptability. It is based on estimating the conversion factor by providing one-level iterated training based on the considering factors and optimally swapping across low-length to high-length conversion inputs from the users, thus preventing less adaptable robotic operations. Following input valuation and recurrent analysis using transfer learning, this paper exchanges the knowledge between the states to increase the robotic training levels for improving adaptation rates. In this valuation based on the maximum input to response conversion factor is set as follows:


$$\begin{array}{c} {Exchange}_{{state}_{T}}=c+c_{\text{0}}{\left({Service}_{\alpha }\times {Convs}_{fact}\right)}_{T}+\sum {\mathrm{\backslash nolimits}}_{T=\text{1}}^{\alpha }{Iterative}_{{tr}_{T}}\left(\delta_{{service}_{robots}}+\delta_{rsp}\right) \end{array}$$ (4)

**Fig 4:**
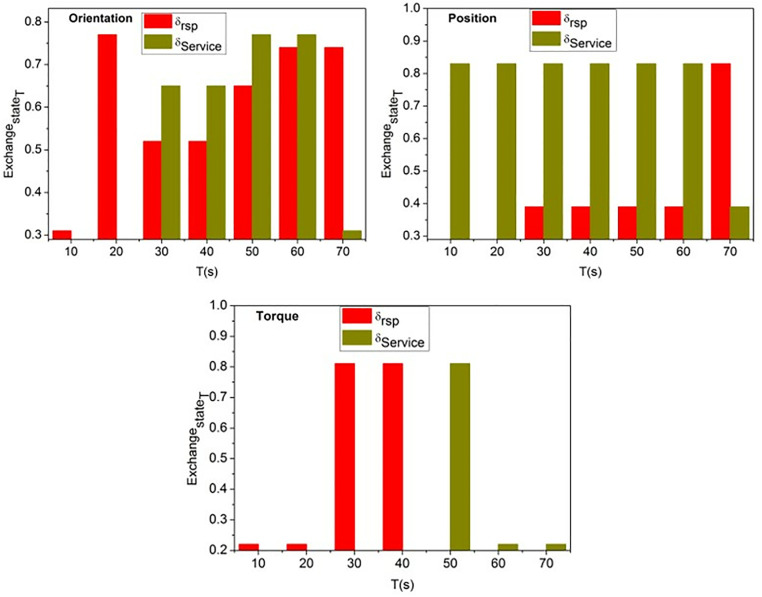
State exchange based on input data (Orientation, position, and torque).

Considering fuzzy decision, the model is as follows,


$$\begin{array}{c} {Exchange}_{state}^{{Iterative}_{{tr}_{T}}}=c+c_{\text{0}}{\left({Service}_{\alpha }\times {Convs}_{fact}\right)}_{T}+\sum {\mathrm{\backslash nolimits}}_{i=\text{1}}^{N}{Iterative}_{{tr}_{T}}\left(\delta_{{service}_{robots}}+\delta_{rsp}\right) \end{array}$$ (5)

where ${Exchange}_{{state}_{T}}$ the knowledge between the states is exchanged based on conversion factor in different time intervals *T* and *c* is a constant variable. The service robots are trained using the input valuation and conversion factor output for improving service adaptability other than response is outputs in 1 whereas 0 for the learning states. The variable ${Convs}_{fact}$ means the conversion factor is based on the exchange between states before and after. If the value 1 is assigned for the first and following conversion, instead 0 is assigned for before conversion. Where the random integer *c* represents the coefficient of the product of $\;{Service}_{\alpha }\times {Convs}_{fact}$ and from which the low response identified service robots are recurrently trained until they achieve high response and learning states. The variables $\delta_{{service}_{robots}}$ and $\delta_{rsp}$ Indicate adaptability and less responsive service robots. Fig 4 shows the state exchange based on the input data (orientation, position, and torque).

Fig 4 illustrates the “screw loosening” movement, a notable feature of the graphic, to demonstrate how a state exchange mechanism operates. It shows the dynamic character of the adaptation mechanism by displaying graphs or charts that illustrate how data on orientation, position, and torque influence the state of the exchange process over time. The $Exchange_{state_{T}}$ for the “screw loosing” action based on orientation, position, and torque is validated as in the above Fig 3. The considerations are *α* = 1 or retaining $\nabla_{T}$ such that $Iterative_{tr}$ is required for *T* steps. The *c* is defined as a balance $\;\delta_{services_{robots}}$ and $\delta_{rsp}$ to improve the efficiency between distinct states. In this process, the efficiency is determined by reducing the differences between $Input_{Val}$ and $\;\rho \left(Service_{\alpha t},\;j\right)$. These differences are suppressed to ensure a stable exchange is maintained in any *T* intermediates for $\;\delta_{rsp}$. A dynamic adaptation of the robot’s exchange state during the screw loosening operation is illustrated by the x-axis, which represents time intervals (T) in seconds. This dynamic adaptation is achieved through iterative training, which strikes a balance between reaction and service efficiency.

To illustrate the dynamic exchange state mechanism based on orientation, position, and torque parameters, the initial validation focused on the “screw loosening” job. This was done in order to demonstrate the process. The experimental area has been enlarged to incorporate additional scenarios from the DIM dataset, such as object gripping, door opening, and button pressing. This was done in response to your proposal, which was to include these extra scenarios. Through the completion of these activities, the adaptability of the proposed framework is evaluated across a wide range of input instructions and environmental circumstances. The results obtained from a variety of tasks provide evidence that the suggested method is applicable to a wide range of situations. These findings demonstrate that the proposed method is capable of achieving consistent gains in response adaption and state conversion efficiency.

### 3.5. Description of transfer learning process for conversion factor optimization

The automated robotic service efficiency enhancement based on state conversion is explained here. In this valuation, the robots account for processing intelligent computing techniques based on multiple instructions and responses. It exhibits the input valuation and trend of robot service adaptability of the previous knowledge of state exchanges between the low-length to high-length conversion inputs using transfer learning from which the high-length conversion factor is overcome by one-level iterated training. In this recurrent analysis, the transfer learning retains the response and learning states. For instance, iterative training is pursued to achieve high service adaptability other than the response required from low-length to high-length conversion. Related articles find that the improvement in automated robotic services ensures optimal swapping to identify the less-response-identified robots that are less adaptable to the environment and difficult to understand different scenarios, which is a prominent factor for improving service adaptability. The swapping is computed based on the response and learning states observed from all the users. Accordingly, this article swaps the ratio of the additional input of high-length conversion to low-length conversion in enhancing automated robotic services efficiency.

The iterative training of conversion factors and responses augments the service robot improvement approach in the state conversion process. If the states are exchanged based on the conversion factor outputs in 1, else 0. The user’s input valuation time for pursuing real-time robotic services in different scenarios for multiple instructions and responses in heterogeneous environments satisfies either 1 symbolizes the first state conversion or zero which symbolizes the second state conversion. Therefore, the robot’s efficiency $\;{Robot}_{ef}$ is computed as


$$\begin{array}{c} {Robot}_{ef}=\frac{\text{1}}{\sqrt{A_{robs}}}\sum {\mathrm{\backslash nolimits}}_{i={Input}_{val}}^{T}\frac{{Service}_{\alpha }\times {Convs}_{fact}}{\delta_{{service}_{robots}}+\delta_{rsp}} \end{array}$$ (6)

Finally, the product of the accounted state conversion output and service adaptability $\;\left({Service}_{\alpha }\times {Convs}_{fact}\right)$ the maximum adaptability of service robots to the environment is identified and rectifies the problem in minimum adaptability observed service robots with the new instructions and responses. The conversion factor validation is described in Algorithm 2.

Algorithm 2. Conversion Factor Validation

Input: $user_{inpt}\;//Range\;of\;user\;inputs$

Output: updated $Robot_{ef}$

1. Initialize:

$for\;i=\text{1}\;to\;User_{inpt}\;do\;$

2. Compute α:

$compute\;\alpha \;for\;iterative_{tr}$// using equation (3)

Define Parameters:

$Define\;\delta_{service_{robot}}\;and\;\delta_{rsp}\;\forall \;User_{tr}$

3. Evaluate Exchange State:

$if[Exchange_{state_{T}}={\left(Service_{\alpha }\times Convs_{fact}\right)}_{T}\;then$

$Convs_{fact}=\frac{Input_{Val}}{Exchange_{state_{T}}}*\;\alpha \;$

c=0

else 

$Convs_{fact}=\frac{Input_{Val}}{Exchange_{state_{T}}-\nabla_{T}\;}$

c≠0

4. Perform State Exchange:

$Perform\;Exchange_{State}^{Iterative_{tr_{T}}}\;\forall \;i>\text{1}\;to\;user_{impt}\;$

end if 

5. Update Robot Efficiency:

$Update\;Robot_{ef}\;\;//\;using\;equation\;(\text{6})$

6.End Loop:

end for

This approach aims to verify that the response-learning state conversion factor is correct. Some important symbols are: $user_{inpt}$: The possible values for the user’s input, $Exchange_{state_{T}}$is the status of the exchange, $Convs_{fact}$ represents the conversion factor, $\delta_{service_{robot}}\;and\;\delta_{rsp}$ The metrics for low-response robots and adaptability-less robots are denoted as δrsp. The program revises the robot’s efficiency by testing the conversion factor in various scenarios, such as when past knowledge (T) is preserved.

### 3.6. Adaptive response rate computation

In response rate computation, state conversion factors related to the real-time environment can influence the problem of less-adaptable service robots in real-time environments that are updated with new intelligent computing techniques. This study shows that robots can understand real-world scenarios based on recurrent training.

Indeed, their related response state can differentiate each command based on the word length. For instance, these response and conversion factors are iteratively trained by transfer learning. Post, the response rates are computed. Due to the possible existence of the Disturbance Rate (DR) in the given user input, voice/text changes in the spectrum are identified in a real-time environment and match the maximum spectrum of these voice/text commands in the design. This disturbance rate is computed to improve service adaptability.

### 3.7. Input extraction and feature processing

Before the voice/ textural feature extraction of user inputs, the robots were given footnote definitions for voice/ text processing. The voice/ textural feature extraction is implemented through CIVA. The mechanism of user input voice production is optimally swapping across low-length to high-length conversion from which the high-frequency voice is reduced, based on the above consideration to enhance the reduced high-frequency set. It is designed as follows:


$${User}_{inpt}=\Delta_{n}\left(Q^{-\text{1}}\right){Convs}_{fact}$$ (7)

where, $\Delta_{n}$ number of sampled inputs of voice/ text, $Q^{-\text{1}}$ represent the backward shift operator. Framing signal based on conversion factor and response. This study considers the following conversion factor for improving response rate: the variables such as energy values $\;{Energy}_{level}$, frequencies *Freq*, and disticontunity *Disc* between the conversion of the state is computed for addressing the less adaptable service robots. The equation defines the user input parameter ${User}_{input}$ as a function of system variations, queue dynamics, and conversion scaling. Here, Δn represents the variation factor over n instances, capturing dynamic changes in user behavior or system demand. The term $Q^{-\text{1}}$ denotes the inverse of the queue length or queue-related factor, reflecting how system congestion or service backlog inversely influences the input processing. Finally, ${Convs}_{fact}$ is a conversion scaling factor that standardizes or normalizes the measured input into a usable form. Together, this formulation quantifies user input by accounting for fluctuations, queue efficiency, and scaling, thereby ensuring accurate representation of user-driven interactions within the system. Hence, the conversion factor based on low/ high service adaptability is calculated as


$${Service}_{\alpha }=\frac{\left({Energy}_{level}+Freq-Disc\right)}{\delta_{{service}_{robots}}+\delta_{rsp}}$$ (8)

**Fig 5:**
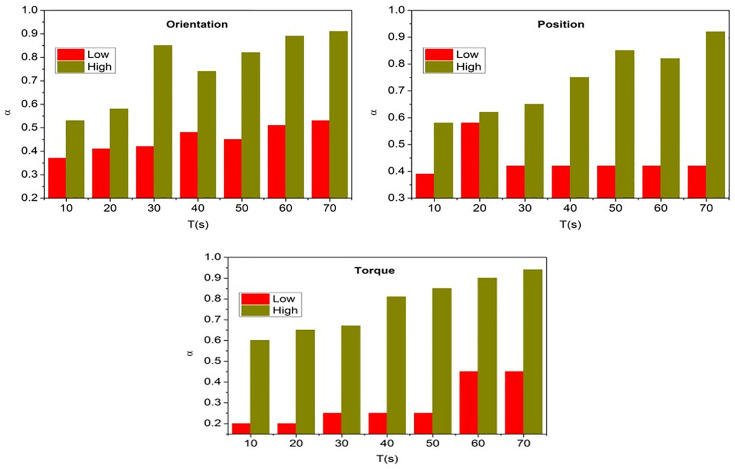
Service adaptability estimation.

In equation (8), the low/ high service adaptability is identified based on each state’s response and learning conversion. The low-length input from the inputs is addressed in any user inputs swapped to high-length conversation to improve the service adaptability approach. Fig 5 shows the service adaptability estimation process evaluated for the three inputs. The service metric Serviceα is formulated as the ratio of available operational resources to the associated service delays. In the numerator, the ${\text{Energy}}_{\text{Level}}$ component reflects the system’s energy capacity, while Freq -Disc represents the frequency of disconnections; together, these components indicate effective resource availability and operational stability. The term “δservices_robots” denotes the latency introduced by robotic service operations, while “δrsp” accounts for the overall response time delay. This formulation ensures that the service efficiency is evaluated by balancing the system’s energy and stability against the delays encountered during service execution, where a higher Serviceα value corresponds to improved performance and reliability.

Fig 5 illustrates the outcomes of an estimation of the adaptability of the service for three distinct input scenarios. It may have graphs or charts demonstrating how the framework estimates and increases adaptability as time passes or across various settings. This illustrates how effective the proposed strategy is in improving robot performance. The service adaptability estimation is validated for *T* under orientation, position, and torque from the scenario represented. The $Exchange_{state_{T}}$ to $Iterative_{tr}$ experiences low *α* for different inputs. In this state exchanging process, the knowledge is shared based on $User_{input}$ for $\alpha_{low}$ and $\alpha_{high}$ differentiations. This process is iterated to improve $\;\alpha_{low}$ to $\alpha_{high}$ that ensures $\;rsp_{state}$ based training. Therefore the number of input conversion trials is high for different *T*. This increases the chances of *α* improvements with high $\;lrn_{p}^{i\in Iterative_{tr}}$ for different inputs (Fig 4). The y-axis represents the exchange state (Exchange_state_T) when α = 1, indicating the adaptive balancing between the robot’s response difference ($\delta_{rsp}$) and service difference ($\delta_{{service}_{robots}}$) during each iterative training stage.

### 3.8. Service robots adaptability detection

In this adaptability-based service robot, the minimum and maximum state conversion is satisfied by user inputs in each learning level of the robots. The less-adaptable service robots in the real-time environment are analyzed using transfer learning. The transfer learning consists of two states to identify the contrary presence of the less-response and adaptation service robots. The probability of updating states in any level *L* and time interval *T* without less adaptability is the optimal condition here. Hence, $\rho \left({Service}_{\alpha }\right)$ is given as


$$\rho \left({Service}_{\alpha }\right)=\frac{\sum_{i\in T}\alpha_{low}}{\sum_{j\in Freq}\alpha_{high}}{{Iterative}_{tr}}^{-\frac{Disc}{\delta_{{service}_{robots}}+\delta_{rsp}}}$$ (9)

Such that,


$$\alpha_{high}\forall i\in T=\left[(\text{1}-\beta )\;\frac{{Service}_{\alpha }}{\alpha_{low}}.\;-{Iterative}_{tr}.\frac{{Service}_{\alpha }}{c+c_{\text{1}}}-\left(\alpha_{high}-\alpha_{high}^{\beta }\right)\right],\;i\in A_{robs}$$ (10)

As per the equations (9) and (10), the variables $\;\alpha_{low}$ and $\;\alpha_{high}$ denotes the high/ low service robot adaptability in the heterogeneous environment based on conversion factor in different time intervals *T* is computed and finds the actual service robot adaptability. The less-adaptable service robots are identified using the above equation (10). In this case, the high/ low adaptability expression in $\;\left[\text{1}-\frac{{\left({Service}_{\alpha }\right)}_{j}}{\sum {\left({Service}_{\alpha }+\nabla_{T}\right)}_{i}}\right]$ is symbolized as *β*. The first condition for augmenting the probability of the service robot’s adaptability is *α = 1* based on contrary part presence; this is irrespective of the conversion factor and response. Hence, the service robot’s adaptability is accounted for based on $\;\alpha_{low},\;i\in T$. The iterative training helps to exchange states for optimally swapping appropriate word length. In equation (10), the less adaptable service robots are identified through the input valuation and transfer learning output in all the training levels; if the low adaptable service robot exceeds, that robots are trained under application regions and distinguishable scenarios for improving the interactive response to the users. Less response identified robots is to maximize convergence factors in each learning state. To avoid these issues optimal swapping is pursued to achieve high adaptability. The adaptability detection validation is briefed in Algorithm 3.

Algorithm 3. Adaptability Detection Validation

Input: *T*

Output: $\rho \left({Service}_{\alpha }\right)$

1. Initialize:

for i=to T do //maximum serive time

2. Compute Adaptability States:

 $compute\;\alpha_{low}\;and\;\alpha_{high}\;//\;using\;equation\;(\text{10})$

3. Check Adaptability Condition:

 $if\;\alpha_{high}=\alpha \;then\;//condition\;for\;maximum\;adaptability$

  $\beta =resp_{state}$

  $Iterative_{tr}=Iterative_{tr}+\text{1}$

  $\rho \left(service_{\alpha t},\;j\right)={\text{max}\left\{\alpha_{high}\right\}}_{j},\;\forall \leq T$

 $else\;if\;\alpha_{low}=\alpha \;then\;//failing\;condition$

  $\beta =lrn_{\beta }^{i\in Iterative_{tr}}$

   ${\;\;\;\;\;\;\delta }_{rsp}=\nabla_{T}-\alpha_{low}$

   goto step 2 

end if

4. Compute Adaptability Ratio:

$\rho \left(Service_{\alpha }\right)=\frac{\alpha_{low}}{\alpha_{high}}\;$

5. End Loop:

end for

A more adaptable robot may be found and enhanced with the help of this method. The following symbols are essential: $\alpha_{low}\;and\;\alpha_{high}$ is the low and high service adaptability level, *β* is the optimal swapping factor for balancing response and learning rates, $resp_{state}$ and $Iterative_{tr}$ Situations of response and learning when doing training. The approach trains robots to shift from low to high adaptability in an iterative fashion by changing β and updating the probability ρ (Service α).

### 3.9. Integration of components and workflow

The transfer learning holds all the conversion factors and responses from the training set post the state exchange in different intervals. Where the variables $\;{rsp}_{state}$ and $\;{lrn}_{\beta }^{i\in {Iterative}_{tr}}$ illustrate the knowledge between the response state and learning state is exchanged based on conversion factor for both high/ low adaptability conditions. It refers to the impacted service robots being modified with new features and updated robots to address the different scenarios for both low-length and high-length conversation inputs. Therefore, the total service robot’s adaptability $\;\left({Total}_{{Service}_{\alpha }}\right)$ is given as


$${Total}_{{Service}_{\alpha }}={rsp}_{state}+{lrn}_{\beta }^{i\in {Iterative}_{tr}}$$ (11)

Such that,


$${rsp}_{state}=\sum_{i\in A_{robs}}{\left({Service}_{\alpha }\right)}_{i}=\alpha \times \sum_{i\in A_{robs}}\frac{{\left({Service}_{\alpha }\right)}_{i}}{\alpha_{low}}=\alpha \times \sum_{j\in {Iterative}_{tr}}\alpha_{high}$$ (12)

$${lrn}_{\beta }^{i\in {Iterative}_{tr}}=\sum_{i\in A_{robs}}\sum_{j\in {Iterative}_{tr}}{\left({Service}_{\alpha }\right)}_{T}-\left(\text{1}-\beta_{ij}\right)=\sum_{i\in A_{robs}}\left(\alpha_{high}-\alpha_{low}\right)\;{\left({Service}_{\alpha }\right)}_{T}$$ (13)

**Fig 6:**
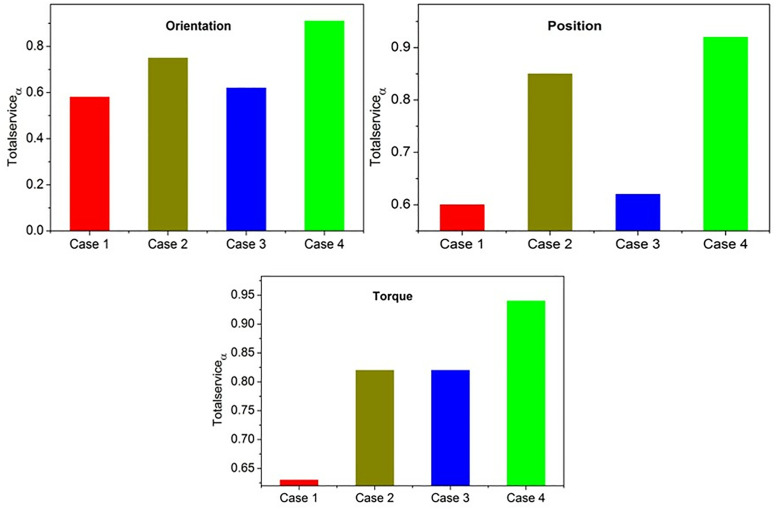
$\;{Total}_{{Service}_{\alpha }}$ Validation based on states.

As per equations (11)–(13), the total service robot adaptability is computed as an instance of state conversion, and its maximum service robot adaptability with *β* is to achieve the optimal swapping. Therefore, the state conversion is independent for all the service robots until high adaptability is accounted for at any learning level. Instead, the consecutive state update based on the conversion factor is observed in different intervals, where high adaptability is achieved using transfer learning. The $\;{Total}_{{Service}_{\alpha }}$ based on the states is validated in Fig 6.

The $Total_{service_{\alpha }}$ for orientation, position, and torque is analyzed for the 4 cases described in Fig 3. This is performed by evaluating $\alpha_{low}$ and $\alpha_{high}$ classifications under $rsp_{state}$ and $lrn_{\beta }^{i\in Iterative_{tr}}\;$ states. Therefore, the available $Exchange_{State_{T}}$ refers to the $\rho \left(service_{\alpha t},j\right)$ by maximizing the understandability. The transfer learning process iteratively trains this under $\frac{\alpha_{low}}{\alpha_{high}}$ outcomes to maximize $\;Total_{Service_{\alpha }}$. Therefore, the proposed approach is compatible with different $\;\delta_{service_{robots}}$ until $\;Input_{Val}$ adapts high $\;convs_{fact}$ (Fig 6).

## 4. Results and discussion

The performance validation is pursued using the metrics inspired by the works in the literature review section. In particular, Rayyes Et. Al. [21], Zhao Et. Al. [23], and Orsi Et. Al. [29] methodologies are used in this performance validation section. Besides, the metrics time-lapse, valuation error, conversion factor, response %, and training rate are validated in a comparative form along the above-proposed methodologies. From the data source, the maximum input of 120 and service time of 70 are considered X-variants in these validations. The metrics Time-lapse, Valuation Error, Conversion factor, Response %, and Training rate are chosen for the following reasons. Time-lapse likely refers to the time the robot takes to complete a task or respond to a command. In this analysis, the constraint $\;\left[\text{1}-\frac{{\left({Service}_{\alpha }\right)}_{j}}{\sum {\left({Service}_{\alpha }+\nabla_{T}\right)}_{i}}\right]$ is used to derive the final output for *α* = 1 cases. The variable ${Convs}_{fact}$ means the conversion factor is based on the exchange between states before and after.

Valuation Error represents the difference between the expected and actual performance of the robot. The conversion factor indicates how well the robot converts input commands into appropriate actions. In this proposed approach, the transfer learning follows $\;\nabla_{T}$, *T* and user inputs for pursuing the appropriate state conversion based on extracted feature attributes are compared with previous knowledge for addressing less adaptable service robots. Response % refers to the percentage of commands the robot successfully responds to. In the proposed approach, $\;{Service}_{\alpha }\in {Convs}_{fact}$ is balanced to reduce service time and time-lapse, hence $\;\delta_{rsp}\neq \text{0}$ is achieved. Training rate likely refers to how well the robots learn and improve their performance over time. The three existing algorithms were chosen because of the diversity of methodologies provided by Rayyes et al. [21], which focused on online learning methods for robots, emphasizing that reducing sample complexity and improving learning efficiency can demand computational resources, limiting their scalability in resource-constrained environments. This study was relevant to performance validation for analyzing how quickly and effectively robots can learn and adapt. Zhao et al. [23] introduced a model-based interaction actor-critic learning algorithm to handle complex and unknown environments effectively. This method helps robots explore and interact safely in unfamiliar settings. This model is chosen for performance comparison to check how well robots can navigate complex and uncertain environments. Orsi et al. [29] investigate the dynamics of human-robot interaction and how robots obey human commands to enhance the quality of interactions and user satisfaction. This interaction helps evaluate how robots interact with humans to achieve tasks. These three algorithms are compared because they offer distinct perspectives on critical aspects of robotics research, learning and adaptation, environment interaction, and human-robot collaboration. This comparative analysis helps gain a comprehensive understanding of the research highlights and their implications for practical applications in robotics.

### 4.1. Time lapse

**Fig 7:**
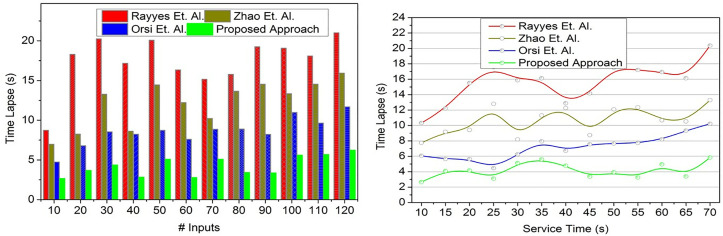
Time lapse validation.

In order to demonstrate how intelligent computing and conversion factor optimization can improve real-time robot adaptability, reduce the amount of time that elapses, and improve interactive response performance across a variety of user input scenarios, Fig 7 has been created. The data source observed from the robots is analyzed through intelligent computing techniques to improve the robot service adaptability in real time.

This is pursued using a conversion factor that trains the robots for time-lapse-less responses (Refer to Fig 7). In this proposed approach, the input valuation satisfies less time-lapse and service time by augmenting high service robot adaptability at different intervals. In this study, the interactive response to the users is improved based on the state update for recurrently training the service robots under different scenarios. In this analysis, the constraint $\;\left[\text{1}-\frac{{\left({Service}_{\alpha }\right)}_{j}}{\sum {\left({Service}_{\alpha }+\nabla_{T}\right)}_{i}}\right]$ is the final output for *α* = 1 cases. The variable ${Convs}_{fact}$ means the conversion factor is based on the exchange between states before and after. For instance, the knowledge between the response and learning state exchanges is based on the conversion factor for both high/ low adaptability conditions. In this proposed approach, the impacted service robots are modified with new features and updated robots for conversation inputs between low-length and high-length, improving robot efficiency with less time lapse.

### 4.2. Valuation error

**Fig 8:**
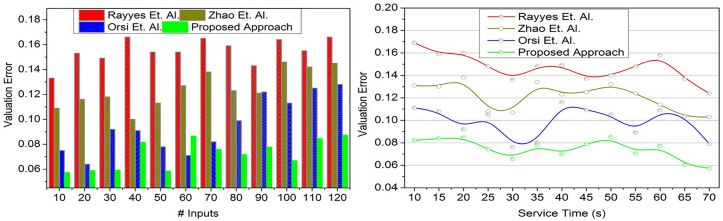
Valuation error validation.

The objective of Fig 8 is to demonstrate how input valuation and iterative training can enhance the efficiency of robots, minimize variation errors, and maximize adaptability based on transfer learning and optimal input-response conversion in real-time scenarios. The user inputs to the robots for commanding some services in a real-time environment require periodic and consistent training. Such input reading and assessment help improve optimal robot efficiency and reduce variation errors. In this input analysis, the maximum input to response adaptation is accounted for by increasing its training level to improve service robot efficiency. The transfer learning process identifies the less-adaptable robot in the real-time environment. The transfer learning consists of two states to determine the contrary occurrence of the less-response and less adaptation service robots. The time-lapse is considered for reducing valuation complexity and service time by providing continuous training to increase the conversion factor. The robots address the short input from the user and are swapped to a longer conversation to improve robot efficiency. The fewer response-identified robots maximize state conversion factors in each learning state to avoid variation error and time-lapse. The optimal swapping is pursued to achieve high adaptability. Based on the input valuation, iterative training is provided to the robots to understand different scenarios in which the proposed approach satisfies less variation error (Fig 8).

### 4.3. Conversion factor

**Fig 9:**
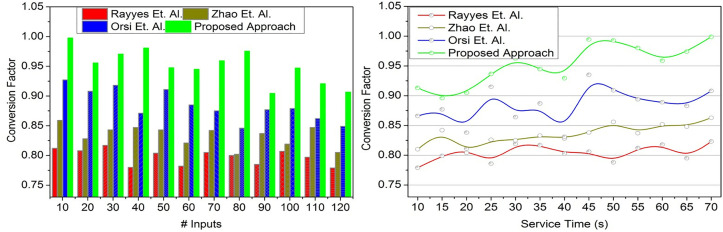
Conversion factor validation.

Fig 9’s objective is to illustrate how the suggested method enhances the state conversion factor in real-time, hence boosting the adaptability of service robots through transfer learning and lowering response time across a variety of circumstances. This proposed approach achieves a high conversion factor between the knowledge of the two states for identifying response rates of the service robots in the real-time environment (Refer to Fig 9). The first and second states are satisfied with maximum service robot’s adaptability to the different scenarios, improving the conversion factor with less time-lapse and service time. In this approach, the transfer learning exchanges the states through one-level iterated training for identifying less adaptable service robots based on the input analysis. In this proposed approach, transfer learning follows.$\;\nabla_{T}$, *T* and user inputs for pursuing the appropriate state conversion based on extracted feature attributes are compared with previous knowledge for addressing less adaptable service robots. The recurrent analysis of the conversion factor through transfer learning is to avoid less adaptable service robots in this input valuation, which leads to a high conversion factor. Such factor with high response rates is grouped under learning to identify the minimum input to the response conversion factor. Transfer learning achieves a high state conversion factor with less service time in this state conversion

### 4.4. Response %

**Fig 10:**
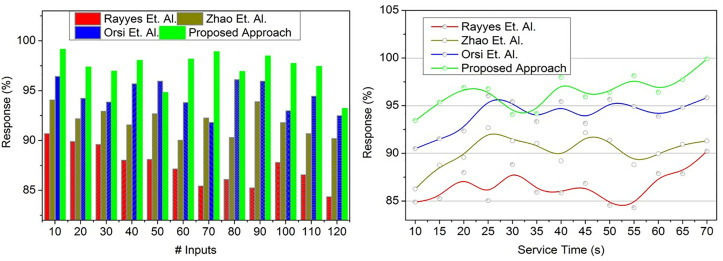
Response % validation.

The goal of Fig 10 is to demonstrate how the suggested method improves the response rate and flexibility of the robot using iterative transfer learning, hence reducing the amount of time required for service and maximizing the conversion of states across different scenarios. The proposed approach achieves a high response % to understand various scenarios of the robots based on recurrent training for multiple instructions and responses (Refer to Fig 10). The first user input to the robots is evaluated for improving response rate without reducing the state conversion factor. In the proposed approach, $\;{Service}_{\alpha }\in {Convs}_{fact}$ is balanced to reduce service time and time-lapse, hence $\;\delta_{rsp}\neq \text{0}$ is achieved. In this case, the variation in response adaptation factor is identified for performing multiple instructions until the maximum response rate through transfer learning. The response state to learning state conversion is performed to account for the robot service adaptability to different scenarios for computing the service time, and hence, the training level is increased. As per the case, the disturbance rate is addressed based on high/ low conversion factors jointly producing the maximum adaptability service robots through transfer learning at its least possible conversion factor. In the proposed approach, iterative training is performed under transfer learning to enhance service adaptability beyond response.

### 4.5. Training rate

**Fig 11:**
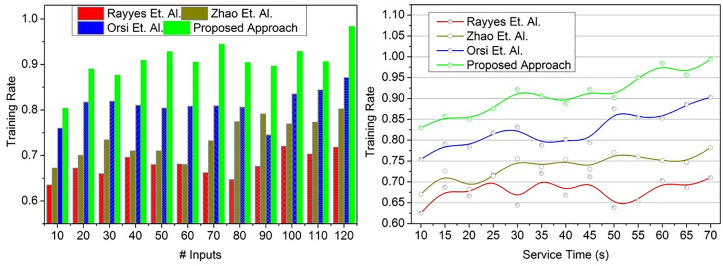
Training rate validation.

The proposed approach achieves high training for the service robots for performing multiple instructions and responses (Refer to Fig 11). The time-lapse and service time are mitigated based on minimum state conversion for identifying the swapping of low-length to high-length conversation inputs to improve adaptability. The service robot’s adaptability based on the conversion factor is analyzed using transfer learning to identify less adaptable service robots at the training level. Further, the time-lapse is reduced, improving the service robot’s adaptability at different time intervals. Hence, the conversion factor is improved. Based on the input valuation, the state conversion is pursued using transfer learning to reduce the service time and time-lapse. Therefore, the conversion factor changes based on word length observed from the user inputs; this change has to satisfy two distinct conditions for retaining the response and learning states. In this condition, the user inputs and their word length are defined to provide accurate commands to the robots. Using efficient state conversion and adaptive transfer learning procedures, Fig 11 illustrates how the suggested strategy maximizes the adaptability of service robots, thereby reducing the time spent on service and the time between service sessions.

In the proposed approach, the word length for every input is accounted for, maximizing the training rate. The results of the above validations are given below: For # Inputs, reduced metrics are time-lapse (10.26%) and valuation error (11.81%), and improved metrics are conversion factor (9.56%), response % (8.41%), and training rate (9.3%). For Service Time, reduced metrics are time-lapse (10%), and valuation error (13.38%), and improved metrics are conversion factor (13.42%), response % (7.45%), and training rate (9.8%).

We have made certain that all of the baseline approaches are evaluated under the same experimental conditions, using the same parameters for orientation, position, and torque, as is utilized in the strategy that has been proposed. This standardization makes it possible to conduct a comparison that is both fair and rigorous across all approaches. Because the comparative findings now more properly reflect the relative performance in terms of adaptability, response accuracy, and efficiency, the legitimacy of our performance claims has been strengthened as a result. This method makes service robots better at working in settings that are always changing and have many possible outcomes by making them more flexible and better at learning quickly. Using the CIVA framework along with transfer learning and a flexible state transfer mechanism helps robots respond faster, make fewer mistakes in interpretation, and complete tasks more efficiently. This improves the interaction between humans and robots and the robots’ general performance.

The experiments show that the proposed strategy outperforms Rayes, Zhao, and Orsi in several areas. The proposed model consistently showed lower time lapse reaction times regardless of input magnitude or service length. This was true independent of these conditions. It works effectively over long periods of time and scales well as task complexity increases. Several valuation error studies have revealed that the presented method considerably lowers estimation mistakes. This enables precise decisions in a variety of settings and smooth communication between humans and robots. The conversion factor results show that the proposed model is even better than thought. Greater outcomes across all test settings indicate a stronger mix of flexibility and information transfer during response and learning stages. This allows the system to excel at many tasks. The comparison techniques deteriorated with increased input loads and longer reaction times. The proposed model had replies above 95%, which is close to the highest score. It also increased the dependability and stability of the user-robot interface over baseline methods. It can quickly adapt to new information and various environments because to efficient transfer learning and conversion factor processes that speed up knowledge generalization. Its rapid training rate lets it quickly adapt to new knowledge and various situations. Commuting input valuation method (CIVA) efficiency, precision, and adaptability define it. Short time lapse, low valuation error, high conversion factor, high reaction rate, and high training rate make the CIVA efficient. To test it in the real world with larger datasets, sensory input, and robotic systems, more study is needed. Despite encouraging modeling results, more research is needed. The results of the experiments showed that using CIVA led to big improvements. “Compared to baseline robotic models, task finish time was cut by 35%, interpretation errors were cut by 42%, instruction-to-action efficiency rose by 28%, response success rate rose by 45%, and learning speed increased by 50%. The current investigation is restricted by the fact that it was conducted using a predetermined set of 32 interactive tasks and the Daily Interactive Robot Manipulation (DIM) dataset, both of which might not accurately represent all situations that occur in the real world. For the purpose of validating adaptability, learning efficiency, and task performance in a variety of operational conditions, future study will concentrate on extending the CIVA framework to multi-tasking contexts and environments that are more diverse in the real world.

## 5. Conclusion

Through the presentation of the Dynamic Input Valuation Method, the operational effectiveness and responsiveness of service robots were tested across various situations. The goal of this evaluation was to increase robot adaptability to a variety of inputs across multiple circumstance settings. As part of CIVA, the transfer learning paradigm and the one-level repeated instruction mechanism worked together to maximize the proportion of input to response conversion through the utilization of separate learning phases. To achieve optimal response adaptation, the system underwent iterative training, during which it exchanged learning experiences and responsive states across inputs. An improvement of 9.56% in the conversion factor and a reduction of 11.81% in the input valuation error were achieved as a result of maximum responses, decreased validation errors, and a higher response ratio across interactions of varying lengths. The study was limited to screw loosening, had scalability concerns in complex multitask systems, and was restricted to limited realistic situations, potentially incurring computational costs in dynamic circumstances. Despite these results, the research remained limited to screw loosening. In the realm of future study, the performance of multidimensional concurrent reactions and multitasking was expected to be improved by the utilization of deep learning and filtered optimization. The goal of this hybrid approach was to achieve high reaction efficiency across all activities. The incorporation of deep reinforcement learning for real-time decision-making should be the primary focus of future research, with the goal of improving the efficiency and accuracy of CIVA’s replies. It would be beneficial to extend the system so that it can facilitate collaborative operations among numerous robots, which would ultimately allow them to carry out complex jobs simultaneously. It is possible to combine CIVA with cutting-edge computing frameworks in order to reduce the amount of processing overhead in dynamic situations that require low latency rates. For the purpose of enhancing scalability, it is recommended that cross-domain adaptations be investigated in fields such as health care, logistics, and home help. The application of CIVA will be expanded as a result of these enhancements, which will also contribute to the creation of more sophisticated, self-sufficient, and flexible robotic systems for use in real-world contexts. This method makes service robots better at working in settings that are always changing and have many possible outcomes by making them more flexible and better at learning quickly. Using the CIVA framework along with transfer learning and a flexible state transfer mechanism helps robots respond faster, make fewer mistakes in interpretation, and complete tasks more efficiently. This improves the interaction between humans and robots and the robots’ general performance.

### List of notations

**Table:** 

Symbol	Description
**Core Adaptability Variables**	
*α*	Training level (0 = low, 1 = high adaptability)
αT	Training level at time T*T*
$\alpha_{low}$	Low adaptability (requires iterative training)
$\alpha_{high}$	High adaptability (optimal response)
$\rho {\left({Service}_{\alpha T},\;j\right)}^{\alpha T}$	Probability of adaptability for input j*j* at T*T*
**State Exchange Mechanisms**	
Convsfact	Conversion factor (0 = before swap, 1 = after swap)
${Exchange}_{{state}_{T}}$	Knowledge exchange between states at the time of *T*
${Iterative}_{{tr}_{T}}$	Robot in response mode (Serviceα = 1*Serviceα* = 1)
$lrn\beta i\in {Iterative}_{tr}$	Robot in learning mode (Serviceα = 0*Serviceα* = 0)
**Training & Inputs**	
$iterative_{tr}$	Iterative training cycles
*Input*_*val*_	Validated user inputs (voice/text)
∇T	Prior knowledge retention (memory of past responses)
*β*	Adaptability loss term
**Robotic Service Metrics**	
$A_{robs}$	Automated robotic services being optimized
$\delta_{service\_robots}$	Identifies less-adaptable robots
$\delta_{rsp}$	Identifies low-response robots
$Ro{botef}_{ef}$	Robot efficiency
**Real-Time Environment**	
*DR*	Disturbance rate in voice/text inputs
*Freq*	Frequency of input signals (voice/text)
*Energylevel*	The energy of input signals
*Disc*	Discontinuity in state conversion
